# Auditory Discrimination of Parametrically Sonified EEG Signals in Alzheimer’s Disease

**DOI:** 10.3390/jcm15010140

**Published:** 2025-12-24

**Authors:** Rubén Pérez-Elvira, Javier Oltra-Cucarella, María Agudo Juan, Luis Polo-Ferrero, Raúl Juárez-Vela, Jorge Bosch-Bayard, Manuel Quintana Díaz, Bogdan Neamtu, Alfonso Salgado-Ruiz

**Affiliations:** 1 Department of Psychobiology, Faculty of Psychology, Pontifical University of Salamanca, 37002 Salamanca, Spain; asalgadoru@upsa.es; 2 Neuropsychophysiology Laboratory, NEPSA Rehabilitación Neurológica, 37003 Salamanca, Spain; mjagudojuan@gmail.com; 3 Department of Health Psychology, Miguel Hernández University, 03202 Elche, Spain; joltra@umh.es; 4 Department of Nursing and Physiotherapy, University of Salamanca, 37007 Salamanca, Spain; pfluis@usal.es; 5 Department of Nursing, Faculty of Health Sciences, University of La Rioja, 26004 Logroño, Spain; raul.juarez@unirioja.es; 6 Faculty of Psychology, Carl von Ossietzky Universität Oldenburg, 26129 Oldenburg, Germany; oldgandalf@gmail.com; 7 Intensive Care Unit, La Paz University Hospital, 28046 Madrid, Spain; 8 Faculty of Medicine, Lucian Blaga University of Sibiu, 550169 Sibiu, Romania; bogdan.neamtu@ulbsibiu.ro; 9 Pediatric Clinical Hospital, 550178 Sibiu, Romania

**Keywords:** sonification, electroencephalogram, Alzheimer’s disease, classification

## Abstract

**Background/Objectives**: Alzheimer’s disease (AD) requires accessible and non-invasive biomarkers that can support early detection, especially in settings lacking specialized expertise. Sonification techniques may offer an alternative way to convey neurophysiological information through auditory perception. This study aimed to evaluate whether human listeners without EEG training can discriminate between sonified electroencephalographic (EEG) patterns from patients with AD and healthy controls. **Methods**: EEG recordings from 65 subjects (36 with Alzheimer’s, 29 controls) from the Open-Neuro ds004504 dataset were used. Data were processed through sliding-window spectral analysis, extracting relative band powers across five frequency bands (delta: 1–4 Hz, theta: 4–8 Hz, alpha: 8–13 Hz, beta: 13–30 Hz, gamma: 30–45 Hz) and spectral entropy, aggregated across 10 topographic regions. Extracted features were sonified via parameter mapping to independent synthesis sources per frequency band, implemented in an interactive web interface (Tone.js v14.8.49) enabling auditory evaluation. Eight evaluators without EEG experience blindly classified subjects into two groups based solely on listening to the sonifications. **Results**: Listeners achieved a mean classification accuracy of 76.12% (SD = 17.95%; range: 49.25–97.01%), exceeding chance performance (*p* = 0.001, permutation test). Accuracy variability across evaluators suggests that certain auditory cues derived from the sonified features were consistently perceived. **Conclusions**: Parametric EEG sonification preserves discriminative neurophysiological information that can be perceived through auditory evaluation, enabling above-chance differentiation between Alzheimer’s patients and healthy controls without technical expertise. This proof-of-concept study supports sonification as a complementary, accessible method for examining brain patterns in neurodegenerative diseases and highlight its potential contribution to the development of accessible diagnostic tools.

## 1. Introduction

Alzheimer’s disease (AD) is the most prevalent form of dementia worldwide, affecting millions of people and posing a significant challenge to public health systems [1]. Population aging and increased life expectancy have intensified the need to develop early and accessible diagnostic tools. Exploring alternative approaches to convey neurophysiological information may contribute to future methodological development in this area [2,3].

Traditionally, the diagnosis of AD has been based on clinical evaluation and neuropsychological testing, supplemented by neuroimaging techniques such as magnetic resonance imaging and positron emission tomography [4,5]. However, these techniques can be costly, invasive, and not always accessible, especially in settings with limited resources.

Electroencephalography (EEG) has emerged as a promising alternative due to its ability to reflect brain electrical activity in real time, its low cost, and its non-invasive nature [6,7]. Recent studies have shown that certain EEG characteristics, such as decreased activity in fast frequency bands and increased activity in slow bands, are associated with the progression of AD [8,9]. In addition, more advanced EEG analyses, such as the assessment of signal complexity and functional connectivity, have shown potential for differentiating between healthy individuals and those with mild cognitive impairment or AD [10,11].

Sonification, defined as the representation of information through sound, has emerged as a promising technique for analyzing complex signals in neuroscience [12,13]. When applied to the analysis of brain electrical activity, this technique capitalizes on the human auditory system’s ability to detect subtle temporal patterns and dynamic relationships that may go unnoticed in conventional visual representations [14].

Initial methods include spectral mapping sonification, which converts specific frequency components into audible tones, and distance mapping sonification, which encodes spatial relationships between electrodes into distinguishable sound patterns [15]. More specialized applications have employed auditory alarms for surgical instruments, model-based systems for analyzing epileptic seizures, and discrete frequency transformations for developing brain–computer interfaces [16].

Previous studies on the use of auditory representations of EEG signals for clinical discrimination have demonstrated the potential of this approach across various neurological conditions. Vialatte et al. [17] developed a sonification method specifically applied to the early detection of AD, showing that trained evaluators were able to discriminate pathological patterns in multichannel EEG recordings. Similarly, Gionfrida and Roginska [18] introduced a parametric sonification approach for AD diagnosis based on spectral features extracted from three brain regions, reporting improved diagnostic accuracy when auditory inspection complemented visual EEG analysis. Vialatte et al. [19] further demonstrated that auditory representations of EEG data could reveal subtle abnormalities associated with the progression from mild cognitive impairment to Alzheimer’s disease.

Collectively, these precedents suggest that sonification can render neurophysiologically relevant information perceptible through the auditory modality; however, most prior work has relied on expert training or hybrid visual–auditory approaches, rather than directly examining the discriminative capacity of purely auditory evaluation by non-experts.

At the same time, direct sonification methods, in which EEG signal amplitudes are linearly mapped to sound pressure levels, have proven particularly valuable for maintaining the temporal fidelity of brain activity [20,21]. Taken together, these findings suggest that sonification represents a viable tool for detecting subtle neurophysiological patterns that may not be evident in conventional visual analyses.

Among sonification methods, parameter mapping sonification stands out for assigning data dimensions to controllable parameters of a sound synthesis model, allowing psychoacoustic principles to be exploited to maximize perceptual discriminability [15]. In the context of EEG signals, this approach has proven effective for the auditory detection of subtle neurophysiological patterns by assigning brain frequency bands to differentiated sound timbres [12,16]. Unlike direct amplitude-to-amplitude mapping, which preserves the exact temporal morphology but produces sounds with limited timbral differentiation, parametric mapping prioritizes the extraction of neurophysiologically relevant features and their transformation into synthesis parameters optimized for human perception.

The objective of this study was to evaluate the feasibility of parametric sonification of EEG as a method for revealing discriminative neurophysiological information associated with AD, exploring whether such information can be perceived through auditory evaluation by individuals with no experience in EEG.

## 2. Materials and Methods

### 2.1. Participants and Data

This study was based on resting EEG data with eyes closed, obtained from the public dataset OpenNeuro ds004504 version 1.0.7 [22]. The dataset comprises EEG recordings from a total of 88 subjects, of whom 65 were selected for the present analysis: 36 patients diagnosed with AD and 29 healthy control subjects, matched for age. The remaining 23 subjects had frontotemporal dementia and were therefore not of interest for this study.

EEG recordings were acquired in the 2nd Department of Neurology at AHEPA University Hospital (Thessaloniki, Greece) using a Nihon Kohden EEG-2100 (Nihon Kohden Corporation, Tokyo, Japón) clinical system with 19 scalp electrodes positioned according to the international 10–20 system and two mastoid references (A1-A2). Data were collected at a sampling frequency of 500 Hz with a sensitivity of 10 μV/mm, a time constant of 0.3 s, and a high-frequency filter of 70 Hz.

The characteristics of the participants are shown in Table 1.

### 2.2. EEG Signal Processing

The data were processed using Python 3.13 with the scientific libraries MNE-Python (version 1.5.0) for EEG handling, NumPy (version 1.24.0), and SciPy (version 1.11.0) for signal processing. The processing pipeline included:

1. 0.5–45 Hz bandpass filtering using Butterworth filters.
2. Re-referencing to Average Reference.
3. Artifact Subspace Reconstruction (ASR) for removal of high variance segments.
4. Independent Component Analysis (ICA) using the RunICA algorithm.
5. Automatic detection and rejection of artifactual components using the ICLabel classifier.

### 2.3. Spectral Analysis and Multichannel Parametric Sonification

A multichannel parametric sonification method was implemented, where the extracted spectral characteristics (relative power per band and spectral entropy) were mapped to real-time sound synthesis parameters using the Tone.js library (version 14.8.49), executed in a web browser using the Web Audio API.

The processing pipeline was implemented in two stages.

*Stage 1: Spectral Analysis and Feature Extraction*

An automated script was developed using the following libraries:

1. MNE-Python (version 1.5.0): for reading and preprocessing EEG files in EDF format, including filtering and re-referencing.
2. NumPy (version 1.24.0): for matrix operations and time series processing using sliding windows.
3. SciPy (version 1.11.0): for estimating spectral power density using the Welch method (scipy.signal.welch).
4. JSON (standard module): for serializing spectral characteristics in a structured format, facilitating data exchange.

This pipeline generated individual JSON files per subject, containing time series of relative power per band (Delta 1–4 Hz, Theta 4–8 Hz, Alpha 8–13 Hz, Beta 13–30 Hz, and Gamma 30–45 Hz) and spectral entropy, aggregated into ten topographic regions.

*Stage 2: Real-Time Parametric Sound Synthesis*

Real-time sonification was implemented using:

- Tone.js (versión 14.8.49): Audio synthesis framework that abstracts the Web Audio API (W3C, 2021), providing oscillators, noise generators, filters, and modulators.
- JavaScript ES6+: For parametric mapping logic and dynamic updating of synthesis parameters.
- Native Web Audio API: for spectral analysis using AnalyserNode (1024-bin FFT) and waveform visualization (1024-sample buffer).

Browser-based architecture ensured cross-platform compatibility without requiring software installation, working correctly in Chrome/Edge, Firefox, and Safari.

### 2.4. Audio Technical Specifications

The audio synthesis was performed in real time within the browser’s audio context (AudioContext), whose sampling frequency is automatically adjusted according to the capabilities of the system’s audio hardware. On most modern computers, this frequency is approximately 48 kHz, although it can vary between 44.1 and 96 kHz.

The architecture of the Web Audio API guarantees low-latency processing thanks to the use of optimized buffers and real-time rendering, without the need to pre-render or store audio files.

### 2.5. Multichannel Synthesis Architecture

Ten independent synthesizers were implemented, one for each topographic region (F-L, F-R, C-L, C-R, P-L, P-R, O-L, O-R, T-L, T-R), each composed of five sound sources corresponding to the brain frequency bands. This architecture allowed selective volume control by region and simultaneous or isolated reproduction of topographic soundscapes.

### 2.6. Band-Timbre Mapping Strategy

Each brain frequency band was assigned to a sound source with distinctive timbral characteristics, selected to maximize perceptual discriminability based on psychoacoustic principles:

Delta (1–4 Hz): Low-frequency sine wave oscillator with a pitch range modulated between 45–95 Hz. The instantaneous frequency was controlled by the relative power of the delta band according to the function:

$$f\delta \left(i\right)=\;45\;+\;50\cdot P\delta \left(i\right)\left[Hz\right]$$

where $P\delta \left(i\right)$ represents the normalized relative delta-band power in window i.

Theta (4–8 Hz): Medium frequency sine wave oscillator (90–170 Hz), analogically modulated:

fθ(i) = 90 + 80·Pθ(i) [Hz]

producing tones in the 90–170 Hz range, perceptible as low-mid tones whose pitch increases with theta activity.

Alpha (8–13 Hz): Oscillator with a triangular waveform in the 180–340 Hz range, incorporating random micro-detuning (±6–20 cents) modulated by spectral entropy to simulate natural variability. This timbre represents states of relaxation and brain rest characteristic of the occipital alpha rhythm.

fα(i)=180+160·Pα(i) [Hz]; detune_α(i)=±(6+14·H(i)) [cents]

Beta (13–30 Hz): Pink noise generator (1/f spectrum, 3 dB/octave decay) filtered by high-pass (cutoff frequency: 600 Hz), with variable frequency amplitude modulation (tremolo):

f_trem,β(i) = 3 + 14·Pβ(i) [Hz]

The tremolo speed increases with beta band power, producing faster vibrating textures.

Gamma (30–45 Hz): High-pass filtered white noise generator (cutoff frequency: 2000 Hz), with fast tremolo:

f_trem,γ(i) = 5 + 20·Pγ(i) [Hz]

#### Neurophysiological Basis of the Band–Timbre Mapping

The design of the parametric mapping is grounded in direct correspondences between established EEG biomarkers in Alzheimer’s disease and quantifiable acoustic parameters.

Increase in Delta (1–4 Hz) and Theta (4–8 Hz) power: This is a well-documented marker. AD is electroencephalographically characterized by an increase in both absolute and relative Delta and Theta power [7,8]. In the sonification, these slow-frequency bands are translated into low-frequency sinusoidal oscillators within the audible range (delta: 45–95 Hz; theta: 90–170 Hz). As a result, the sonifications of patients with AD exhibit greater acoustic energy in the low-frequency components. This change is objectively quantifiable through spectral analysis, which reveals a downward shift in the spectral centroid toward lower frequencies. The sonification is coherent because it translates a well established EEG phenomenon in AD into an acoustic correlate that is directly interpretable by the human auditory system. These increases in slow-frequency activity reflect a generalized deceleration of cortical dynamics. By mapping these bands onto low-frequency sound oscillators within the audible range, the increase in slow activity becomes expressed as a greater presence of low-frequency components in the resulting sound. Acoustic validation confirmed this correspondence: AD sonifications showed significantly greater energy in the low-frequency bands (energy < 100 Hz: 33.35 ± 8.49% vs. 27.50 ± 6.07%, *p* = 0.004; energy 90–170 Hz: 27.74 ± 9.51% vs. 15.79 ± 4.62%, *p* < 0.001; Cohen’s d = 1.55), demonstrating that the established neurophysiological biomarker translates directly into a quantifiable acoustic feature (see Section 3.4).

The reduction in alpha (8–13 Hz) and beta (13–30 Hz) power in Alzheimer’s disease is also a well-documented biomarker. The attenuation of fast cortical rhythms correlates with cognitive decline [10,11]. In the sonification, mapping the alpha band to a triangular oscillator (180–340 Hz) and the beta band to a filtered pink-noise generator (high-pass > 600 Hz) produces a quantifiable acoustic outcome: a reduction in spectral content in the mid–high frequency ranges (180–800 Hz), measurable as a decrease in the spectral centroid and a reduction in energy in bands above 500 Hz. The use of triangular waveforms for the alpha band, with their characteristic harmonic richness, and pink noise for the beta band, which provides a dense but non-tonal texture, renders these components perceptually distinct from the pure sine waves used to represent slow activity. This distinction facilitates their discrimination by listeners. This sonification is coherent because it translates the loss of fast cortical rhythms, a characteristic feature of AD, into a perceptible reduction in high-frequency acoustic components. In EEG, the decrease in alpha and beta power reflects a deterioration of rapid cortical dynamics; by mapping these bands onto oscillators and sound sources located in the mid–high region of the audible spectrum, their attenuation manifests as a loss of energy in the higher frequencies. The resulting auditory output, darker and with reduced presence in the upper bands, constitutes a direct and quantifiable acoustic analog of the decline in fast brain rhythms, thereby preserving the underlying neurophysiological logic. This was objectively verified through spectral analysis: AD sonifications exhibited significantly reduced energy in the alpha-mapped band (180–340 Hz: 12.29 ± 7.53% vs. 21.22 ± 6.92%, *p* < 0.001, d = −1.23) and beta-mapped band (600–2000 Hz: 3.99 ± 1.36% vs. 5.68 ± 0.89%, *p* < 0.001, d = −1.43), with an overall spectral centroid shift toward lower frequencies (4062 ± 995 Hz vs. 4822 ± 868 Hz, *p* = 0.004, d = −0.81). These acoustic differences provide objective evidence that the attenuation of fast cortical rhythms in AD is preserved as perceptible reductions in mid-high frequency content (see Section 3.4 and Table 2).

The reduction in gamma activity (30–45 Hz) is a documented finding in AD and other neurodegenerative disorders [23,24]. In the sonification, the gamma band is represented by a white-noise generator processed through a high-pass filter with a cutoff frequency of 2000 Hz. This component is further modulated using a rapid tremolo. This implementation translates gamma oscillations into acoustic content rich in high-frequency aperiodic energy (>2000 Hz), accompanied by fast temporal fluctuations. The resulting auditory output is a quantifiable texture perceptually analogous to the high-frequency bands in audio. The reduction in gamma power in AD thus manifests as a loss of this sonic component in the corresponding spectral region, providing a direct analog of the decrease in fast cortical rhythms. This sonification is clearly distinguishable from lower-frequency components. The strategy preserves the underlying neurophysiological logic: just as the reduction in gamma oscillations reflects a deterioration of high-speed cortical synchronization [25], in the acoustic domain it is expressed as diminished energy in the uppermost spectral regions, reducing the vividness of the gamma component and enabling a direct parallel between brain dynamics and auditory perception. Objective measurement confirmed reduced high-frequency content in AD sonifications (energy > 2000 Hz: 31.43 ± 7.13% vs. 36.74 ± 6.78%, *p* = 0.005, d = −0.76), validating the acoustic manifestation of decreased gamma power (Section 3.4).

Table 2 summarizes the neurophysiological basis of the parametric mapping and presents empirical validation through objective acoustic measurements (see Section 3.4 for full results).

**Table 2:** Neurophysiological Basis of Band-Timbre Mapping and Objective Acoustic Validation.

EEG Biomarker in AD	Change Direction	Synthesis Parameter	Acoustic Range	Quantifiable Acoustic Descriptor	Measured Values (AD vs. Control)	Reference
Delta power	↑	Sine-wave oscillator gain	45–95 Hz	Spectral energy < 100 Hz	33.35 ± 8.49% vs. 27.50 ± 6.07% (*p* = 0.004 **)	Jeong, 2004 [7]
Theta power	↑	Sine-wave oscillator gain	90–170 Hz	Spectral energy 90–170 Hz	27.74 ± 9.51% vs. 15.79 ± 4.62% (*p* < 0.001 ***)	Jeong, 2004 [7]
Alpha power	↓	Triangular-wave oscillator gain	180–340 Hz	Spectral energy 180–340 Hz	12.29 ± 7.53% vs. 21.22 ± 6.92% (*p* < 0.001 ***)	Rossini et al., 2006 [10]
Beta power	↓	Filtered pink-noise gain	>600 Hz	Spectral energy > 600 Hz	3.99 ± 1.36% vs. 5.68 ± 0.89% (*p* < 0.001 ***)	Stam et al., 2003 [11]
Gamma power	↓	Filtered white-noise gain	>2000 Hz	Spectral energy > 2000 Hz	31.43 ± 7.13% vs. 36.74 ± 6.78% (*p* = 0.005 **)	Dauwels et al., 2010 [8]
Spectral entropy	↑	Bit-crushing depth	0–100%	THD	1.15 ± 0.49 vs. 0.77 ± 0.20 (*p* < 0.001 ***)	Horvath et al., 2018 [26]
Spectral entropy	↑	Band-pass filter Q-factor	Q = 1–11	Spectral selectivity (–3 dB bandwidth)	Not directly measured (see Note)	Horvath et al., 2018 [26]
Spectral entropy	↑	Tremolo depth (noise)	0–100%	Temporal amplitude coefficient of variation	0.312 ± 0.045 vs. 0.354 ± 0.035 (*p* < 0.001 ***)	Horvath et al., 2018 [26]

Note: THD = Total Harmonic Distortion. Measured values represent mean ± standard deviation from objective acoustic analysis of generated sonifications (n = 36 AD, n = 29 Control). *p*-values are FDR-corrected (Benjamini–Hochberg). ** *p* < 0.01, *** *p* < 0.001. Full acoustic validation results are presented in Section 3.4 and Table 5. The band-pass filter Q-factor effect on spectral selectivity was not directly measured in this validation but is implicitly reflected in the spectral bandwidth measurements. Q = filter quality factor.

### 2.7. Dynamic Control of Synthesis Parameters

Spectral entropy quantifies the degree of disorganization and complexity of the EEG power spectrum. In Alzheimer’s disease, spectral entropy typically increases due to the loss of well-defined dominant oscillatory rhythms, the flattening of the spectrum through power redistribution, and the rise in irregular broadband activity [26]. Elevated entropy values reflect a loss of functional cortical organization and neuronal desynchronization.

In our system, entropy modulates synthesis parameters that introduce acoustic complexity and variability, establishing a direct correspondence between neuronal disorganization (as quantified by EEG entropy) and perceptual complexity/roughness (as quantified by acoustic descriptors):

1. Source gain per band: Relative powers *Pb*(*i*) were mapped to linear gains using perceptual compression:
  $$g_{b\left(i\right)}={\left[P_{b\left(i\right)}\right]}^{0.85}$$
  where the exponent 0.85 reduces the dynamic range to improve the auditory intelligibility of subtle variations.

2. Regional bandpass filter Q factor: Each region incorporated a bandpass filter centered at 1000 Hz whose quality factor Q (bandwidth) was modulated inversely with the normalized spectral entropy H(i):

Q(i)=1+10·[1−H(i)]

This modulation produces greater resonance and frequency selectivity when entropy is low (ordered spectrum), and lower selectivity when entropy is high (complex spectrum).

3. Entropy-modulated nonlinear processing: Each regional synthesizer incorporated a dynamic crossfade between a clean channel and a channel processed by bit crushing (reducing the bit depth to 4 bits), controlled by entropy：
  $$fade\left(i\right)=0.15+0.85\cdot H\left(i\right)$$
  where fade = 0 corresponds to the clean channel and fade = 1 to the distorted channel. This processing introduces nonlinear harmonics that are perceptible when spectral complexity increases.

4. Tremolo depth in noise sources: The amplitude modulation depth of the beta and gamma sources was directly controlled by entropy:
  $$d_{\beta \left(i\right)}=0.1+0.8\cdot H\left(i\right)$$
  $$d_{\gamma \left(i\right)}=0.15+0.8\cdot H\left(i\right)$$
  producing more pronounced modulations in states of high entropy.

### 2.8. Signal Processing Chain

The architecture of each regional synthesizer followed the following processing chain:

1. Band sources: Five independent sound generators (tonal oscillators for delta/theta/alpha, filtered noise generators for beta/gamma).
2. Band gain control: Dynamic modulation according to relative power levels.
3. Pre-filter mixing bus: Weighted sum of the five sources.
4. Clean/distorted split: Clean channel and channel with bit-crushing in parallel.
5. Dynamic crossfade: Entropy-modulated mixing.
6. Regional bandpass filter: Fixed center frequency (1000 Hz), Q modulated by entropy.
7. Regional gain control: −6 dB attenuation for focused region, −24 dB for non-focused regions (interactive user control).
8. Master bus: Final mix of the 10 regions connected to the audio output.

### 2.9. Temporal Updating and Transition Smoothing

The synthesis was updated synchronously with the advancement of the spectral analysis windows (1 s step). To avoid audible discontinuities (clicks, pops) due to abrupt parameter changes, all transitions were implemented using 80-millisecond exponential ramps, which are short enough to follow rapid dynamics but long enough to ensure perceptual continuity.

The audio sampling rate was automatically determined by the browser’s Web Audio API context (typically 48 kHz), ensuring adequate temporal resolution for the synthesis of high-frequency (gamma) components.

### 2.10. Psychoacoustic Justification of the Approach

This parametric sonification method contrasts with direct amplitude-to-amplitude mapping approaches, in which temporal samples of the EEG signal are linearly converted into sound pressure levels. While direct mapping preserves the exact temporal morphology, it has significant perceptual limitations:

1. Inaudible frequency range: Slow brain waves (delta: 1–4 Hz, theta: 4–8 Hz) are infrasonic or barely audible, requiring extreme temporal compression for perception, which destroys the original temporal correspondence.
2. Limited timbral discriminability: Differences between frequency bands in direct mapping manifest themselves only as variations in amplitude, which are difficult to discern aurally.
3. Sensitivity to artifacts: Direct mapping amplifies high-amplitude artifacts (eye and muscle movements) that can mask subtle neurophysiological patterns.

In contrast, the parametric mapping used in this study exploits principles of timbral discrimination in the human auditory system [27,28], assigning brain frequency bands to sound sources with distinctive spectral characteristics. This strategy enables differences in EEG spectral distribution between Alzheimer’s patients and healthy controls to be translated into global timbral patterns characterized by quantifiable acoustic properties, for example, a downward shift in the spectral centroid and increased aperiodic high-distortion components versus higher mid-high spectral energy and more stable, low-variability amplitude envelopes, respectively.

The elimination of temporal correspondence sample by sample, in favor of maximizing perceptual discriminability through timbral differentiation, a characteristic of this metho, is appropriate, given that the alterations typical of AD manifest themselves mainly in the spectral domain.

The elimination of temporal correspondence sample by sample, in favor of maximizing perceptual discriminability through timbral differentiation (characteristic of this method), is appropriate, given that the characteristic alterations of the EEG in AD are more evident in the spectral domain than in specific transient morphological characteristics [10,29].

The parametric approach has proven effective in previous EEG sonification applications, including detection of epileptic seizures [12], brain–computer interfaces [16], and exploratory analysis of neurophysiological patterns [13], validating its applicability to the auditory analysis of electroencephalographic signals.

### 2.11. Spectral Analysis and Feature Extraction

A sliding-window analysis was implemented in which each segment had a duration of 2 s with 50% overlap (corresponding to a 1 s step). For each window, the power spectral density was estimated using Welch’s method, from which relative power values were derived for the delta (1–4 Hz), theta (4–8 Hz), alpha (8–13 Hz), beta (13–30 Hz), and gamma (30–45 Hz) frequency bands. In addition, normalized spectral entropy was computed as an index of signal complexity.

### 2.12. Objective Acoustic Validation of Sonifications

To empirically validate that differences in EEG biomarkers translate into objectively quantifiable acoustic differences, an acoustic analysis of the generated sonifications was performed.

For each subject, a 48 kHz mono WAV file was generated from the corresponding JSON feature file using the same parametric synthesis pipeline described in Section 2.6. The synthesis was implemented offline in Python 3.13 using NumPy and SciPy to replicate the exact audio processing logic of the web application (Tone.js), ensuring faithful reproduction of the browser-based sonifications.

The following quantifiable acoustic descriptors were extracted from each sonification using the Librosa library (version 0.10.1) [30]:

- Spectral centroid (Hz): Center of mass of the spectrum, indicating perceptual “brightness”;
- Spectral energy distribution: Percentage of total energy within specific frequency bands (45–95 Hz, 90–170 Hz, 180–340 Hz, 600–2000 Hz, >2000 Hz), corresponding to the mapped EEG frequency bands;
- Temporal coefficient of variation: Standard deviation divided by mean of the root-mean-square (RMS) amplitude envelope, quantifying temporal stability;
- Spectral entropy: Shannon entropy of the normalized power spectrum, measuring acoustic complexity;
- Harmonicity estimate: Ratio of low-frequency energy (<100 Hz) to high-frequency energy (>2000 Hz), as a proxy for total harmonic distortion (THD).

### 2.13. Generating JSON Files for Interactive Visualization

The spectral characteristics extracted by windows and aggregated by regions were exported to individual JSON (JavaScript Object Notation) files per subject. The choice of JSON format was based on its native compatibility with web applications and its hierarchical data structure, which allows multidimensional time series to be stored efficiently [31].

Each JSON file contains the following information: subject identification (anonymous participant code), acquisition parameters (original sampling frequency, window duration, and time step), time series (vector of time stamps corresponding to each analyzed window), and characteristics by region: for each of the 10 topographic regions, time-synchronized arrays are included containing the relative powers in the five frequency bands (delta, theta, alpha, beta, gamma) and the normalized spectral entropy (H).

This format allows the web interface to directly access temporal and spectral characteristics without the need for additional processing, facilitating real-time sound synthesis with synchronized updating of the dynamic mapping of spectral characteristics to synthesis parameters using the Tone.js framework [32]. Structuring by regions also allows for the selective activation of topographic sound sources during auditory assessment.

The conversion to JSON was performed using Python’s 3.13 json module, using standard serialization with UTF-8 encoding. The average size of the generated JSON files was 2.4 ± 0.6 MB per subject, depending on the length of the clean EEG recording available after artifact removal.

### 2.14. Aggregation by Topographic Regions

The EEG channels were grouped into 10 topographic regions based on anatomical nomenclature:

- Frontal: F-L (left frontal), F-R (right frontal);
- Central: C-L (left central), C-R (right central);
- Parietal: P-L (left parietal), P-R (right parietal);
- Occipital: O-L (left occipital), O-R (right occipital);
- Temporal: T-L (left temporal), T-R (right temporal).

The midline channels (Cz, Pz, Fz, Oz) were distributed equally between the left and right hemispheres of their respective regions.

### 2.15. Hearing Assessment Interface

An interactive web application was developed in HTML [33] with the following functionalities: loading of individual JSON files per subject, playback of sonifications by independent topographic regions, differential volume control per region through cursor interaction, integrated listening option for all regions simultaneously, and an intuitive interface for navigation between subjects.

### 2.16. Protocol for Evaluating Sonifications

Eight members of the laboratory with no prior experience in EEG interpretation participated as evaluators. They were given the following instructions:

1. “Load individual JSON files by subject in the HTML web application.”
2. “Play sonifications by independent topographic regions or in general.”
3. “Control differential volume by region through cursor interaction, if desired.”
4. “Evaluate each subject based solely on auditory characteristics and place them in a group: Alzheimer’s or Control.”

The evaluation was conducted under single-blind conditions, where the evaluators were unaware of both the subjects’ actual diagnosis and the specific objectives of the study.

### 2.17. Statistical Analysis

The following performance metrics were calculated for each evaluator: accuracy, defined as the proportion of correct classifications out of the total; sensitivity (recall or true positive rate), which indicates the ability to correctly identify cases of Alzheimer’s; specificity (true negative rate), which measures the ability to correctly identify healthy controls; positive predictive value (PPV), which represents the probability that a subject classified as having AD actually has the disease; and negative predictive value (NPV), which indicates the probability that a subject classified as a healthy control does not have the disease.

The statistical significance of each individual evaluator’s performance was assessed using a two-tailed binomial test, comparing the number of correct classifications observed against the level expected by chance. We adopted a conservative chance level of 50.58%, corresponding to the expected accuracy if evaluators responded proportionally to the class frequencies in the dataset (36 Alzheimer’s cases vs. 29 controls), a phenomenon known as probability matching [34]. This threshold is more conservative than the standard uniform random-guessing baseline of 50.00% [35,36] and makes it more difficult to reject the null hypothesis. Given that the difference between both levels is statistically trivial (0.58 percentage points, <0.1 standard errors), the statistical conclusions remain unchanged regardless of this choice. The Holm correction [37] was applied to control the type I error rate in the context of multiple comparisons, ensuring robust control of the family error.

The significance of the overall performance of the group of evaluators was assessed using a nonparametric permutation test. In each iteration, the diagnostic labels were randomized, keeping the structure of evaluators and subjects constant, and the average accuracy of the group was calculated. The *p*-value was estimated as the proportion of permutations that produced an average accuracy equal to or greater than that observed empirically. This nonparametric method is particularly robust in the face of deviations from normality and limited sample sizes.

Inter-rater agreement was quantified using Fleiss’ Kappa coefficient [38], which generalizes Cohen’s Kappa for situations with more than two raters and allows agreement to be assessed beyond the level expected by chance. The 95% confidence intervals for the Kappa coefficient were estimated using nonparametric bootstrapping with 1000 iterations.

The statistical power of the study was estimated using Monte Carlo simulation, modeling the distribution of accuracies under the alternative hypothesis of effective discrimination. This estimate considers the sample size (n = 65 subjects), the number of evaluators (n = 8), the significance level (α = 0.05), and the observed effect.

All statistical analyses were performed using Python 3.13 with the scientific libraries SciPy (version 1.11.0) for statistical tests, NumPy (version 1.24.0) for matrix operations, statsmodels (version 0.14.0) for calculating Kappa and its confidence intervals, and scikit-learn (version 1.3.0) for classification metrics. The significance level was set at α = 0.05 for all statistical tests.

## 3. Results

### 3.1. Classification Analysis

The eight evaluators with no prior experience in EEG interpretation completed the task of classifying the 65 subjects (36 with AD, 29 healthy controls) based solely on auditory perception of the EEG sonifications. The detailed results per evaluator are presented in Table 3.

The individual accuracy analysis showed considerable variability among evaluators. The average accuracy was 76.12% (SD = 17.95%, 95% CI = [60.7%, 86.3%]), ranging from 49.25% to 97.01%. To assess the statistical significance of these results, a binomial test was applied to each evaluator, comparing their performance against the expected random level (50.58%, adjusted for class imbalance: 36 vs. 29) [39].

Six of the eight evaluators obtained statistically significant accuracies after applying Holm’s correction for multiple comparisons (*p* < 0.05). Two evaluators (4 and 5) did not reach statistical significance (*p* = 0.596 and *p* = 0.221, respectively), indicating that their performance did not consistently exceed the level expected by chance.

Sensitivity values (ability to correctly identify cases of AD) ranged from 47.22% to 97.30%, while specificity (ability to correctly identify healthy controls) ranged from 55.17% to 100%. Notably, three evaluators (6, 7, 8) achieved perfect specificity of 100%, indicating that they did not make any false positives. PPV ranged from 56.67% to 100%, and NPV ranged from 45.71% to 96.67%.

To confirm that the overall performance of the group of evaluators was statistically superior to chance, a nonparametric permutation test was performed. The result indicated that the probability of obtaining an average accuracy equal to or greater than 76.12% by pure chance is *p* = 0.001, robustly confirming that auditory discrimination was significantly higher than the expected level. The statistical power analysis revealed a value of 0.916, indicating that the study had sufficient power (>80%) to detect the observed effect, thus minimizing the risk of type II error.

### 3.2. Inter-Evaluator Consistency Analysis

The degree of agreement among the eight independent evaluators was assessed using Fleiss’ Kappa coefficient for categorical variables with multiple evaluators. The overall results are presented in Table 4.

Fleiss’ Kappa coefficient was κ = 0.511 (95% CI = [0.370, 0.636]), corresponding to a moderate level of agreement according to the interpretation scale of Landis and Koch [40], where values between 0.41–0.60 are considered moderate. This result indicates that the agreement between evaluators was substantially higher than expected by chance (κ = 0), but shows sufficient variability to suggest that the evaluators applied partially heterogeneous perceptual criteria.

Of the 65 subjects evaluated, 61 items were used to calculate the Kappa coefficient. The four excluded items correspond to cases in which at least one evaluator was unable to complete the classification due to technical or procedural reasons, specifically audio loading errors or interface issues that prevented data collection. The exclusion rate was low (6.2% of items), and the excluded cases were distributed across both diagnostic groups (2 AD, 2 Control), indicating no systematic bias toward removing perceptually ambiguous items from either category. This exclusion criterion was applied uniformly to ensure balanced data structure as required by Fleiss’ κ methodology.

This moderate level of agreement is a central finding that provides important insight into the nature of the perceptual discrimination task. Moderate agreement (κ = 0.511) indicates that evaluators identified shared, consistent patterns in the sonifications while also exhibiting individual variability in their classifications. Importantly, this level of agreement is both expected and appropriate for complex perceptual tasks. Comparative context from the literature on inter-rater reliability shows that our κ value falls within the typical range for established diagnostic and perceptual tasks, including cardiac auscultation by trained cardiologists (κ = 0.30–0.50; [41]) and vocal emotion recognition (κ = 0.45–0.65; [42]). This comparison demonstrates that the auditory discrimination of EEG sonifications involves a level of perceptual complexity comparable to validated clinical tasks.

The combination of moderate inter-rater agreement with highly significant group-level performance (mean accuracy 76.12%, *p* = 0.001) indicates that discriminative information is objectively present in the sonifications (as validated in Section 3.4) and perceptually accessible to human listeners, but that exploiting this information requires perceptual acuity that varies across individuals. The observed heterogeneity reflects genuine individual differences in auditory sensitivity, perceptual strategies, and attention to temporal versus spectral characteristics of the sonifications—a pattern entirely consistent with the literature on complex auditory discrimination tasks.

### 3.3. Evaluation Time

The average time required per evaluator to complete the classification of the 65 subjects was 108.75 min on average, which represents approximately 1 min and 40 s per evaluation until the classification was decided. This time includes familiarization with the interface and classification decision-making.

### 3.4. Objective Acoustic Validation: Sonifications Reflect EEG Biomarkers

Acoustic analysis confirmed that neurophysiological differences between groups translate into objectively measurable acoustic differences. Table 5 summarizes the key findings.

**Figure 1:**
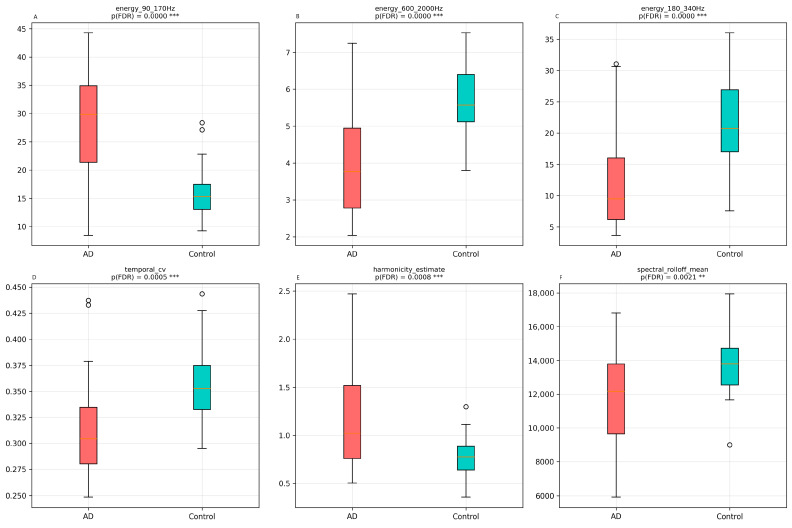
Objective acoustic differences between AD (red) and control (blue) sonifications. Box plots show distributions of (**A**) energy in the 90–170 Hz band (theta mapping), (**B**) energy in the 600–2000 Hz band (beta mapping), (**C**) energy in the 180–340 Hz band (alpha mapping), (**D**) temporal coefficient of variation, (**E**) harmonicity estimate, and (**F**) spectral roll off mean. All comparisons are statistically significant after FDR correction (*** *p* < 0.001, ** *p* < 0.01).

Sonifications from AD patients exhibited significantly greater energy in the 90–170 Hz band (theta mapping, +11.95%, *p* < 0.001, d = 1.55), directly reflecting the established increase in theta power in AD [7,8]. Conversely, they showed reduced energy in the 180–340 Hz (alpha mapping, −8.92%, *p* < 0.001, d = −1.23) and 600–2000 Hz bands (beta mapping, −1.69%, *p* < 0.001, d = −1.43), consistent with the well-documented reduction in alpha and beta power in AD [10,11] (see Figure 1).

In other hand, AD sonifications exhibited a significantly lower spectral centroid (4062 ± 995 Hz vs. 4822 ± 868 Hz, *p* = 0.004, d = −0.81), indicating an overall shift toward lower frequencies. This acoustic feature directly corresponds to the spectral slowing characteristic of AD, where increased slow-wave activity (delta/theta) and decreased fast rhythms (alpha/beta) produce a spectral distribution weighted toward lower frequencies.

Counter to expectations based on increased EEG entropy in AD [43], AD sonifications exhibited lower temporal amplitude variability (CV = 0.312 vs. 0.354, *p* < 0.001). This apparent paradox reflects the dominance of stable low-frequency oscillators (delta/theta) in the synthesis output, whose amplitude envelopes are intrinsically less variable than high-frequency noise components (beta/gamma), which are more prominent in control sonifications.

These results demonstrate that the parametric mapping successfully translates established EEG biomarkers into quantifiable acoustic differences, providing an objective foundation for the perceptual discrimination observed in the listening task (Section 3.1).

## 4. Discussion

The results obtained demonstrate that parametric sonification of EEG spectral characteristics, aggregated by topographic regions, preserves discriminative neurophysiological information that can be perceived auditorily by evaluators without prior experience in electroencephalographic interpretation. The discrimination capacity of 76.12% suggests that spectral differences between Alzheimer’s patients and healthy controls effectively translate into perceptible timbral differences when mapped to psychoacoustically optimized sound synthesis parameters. The classification accuracy of 76.12% represents a significant finding that exceeds the expected level of chance and falls within the range of accuracy reported for conventional EEG biomarkers in the literature [26,44].

A critical concern in auditory display of biosignals is whether perceptual differences reflect genuine physiological differences or merely arbitrary mapping artifacts. Our objective acoustic analysis (Section 3.4) addressed this concern by demonstrating that sonifications preserve the spectral signature of EEG biomarkers. The observed acoustic differences, increased energy in the 90–170 Hz band (+11.95%, *p* < 0.001), reduced energy in the 180–340 Hz band (−8.92%, *p* < 0.001), and downward spectral centroid shift (−760 Hz, *p* = 0.004), are direct acoustic manifestations of the established AD biomarkers: increased theta power, reduced alpha power, and overall spectral slowing [7,8,10].This objective validation strengthens the interpretation that the 76.12% discrimination accuracy achieved by listeners reflects genuine sensitivity to neurophysiological patterns rather than spurious acoustic cues.

To contextualize these findings within the current landscape of electroencephalographic biomarkers, Table 6 presents a systematic comparison of the diagnostic accuracy reported for different methodological approaches. As can be seen, the accuracy of 76.12% obtained through parametric sonification falls within the performance range of established methods, which include quantitative spectral analysis (70–85%; [8]), functional connectivity analysis (75–90%; [10,11]), machine learning techniques (78–92%; [44]), and signal complexity analysis (72–88%; [26]).

While Table 6 situates our findings within the broader landscape of EEG-based approaches for AD detection, most prior work on EEG sonification for neurological diagnosis has focused primarily on epilepsy rather than neurodegenerative disorders. For example, Loui et al. [45] showed that non-expert listeners achieved 64.3% accuracy in identifying epileptic seizures from sonified EEG after minimal training (1 min), starting from near-chance baseline performance (53.1%). Similarly, Frassineti et al. [46] reported that auditory detection of non-motor epileptic seizures using real-time sonification reached accuracy levels comparable to post-processed automatic detection methods. In the specific domain of Alzheimer’s disease, Vialatte et al. [17] applied bump sparse sonification to the early detection of AD, although their approach required trained evaluators. More recently, Gionfrida and Roginska [18] developed a three-tone sonification technique for AD diagnosis, using PET/CT scans rather than EEG, and reported accuracy improvements of 10–30% when auditory feedback was added to visual inspection by radiologists (baseline: 50–55%; with sonification: 56–88%, depending on disease severity). However, their method was specific to PET imaging and required prior radiological expertise. In contrast, our parametric EEG sonification approach achieved a mean accuracy of 76.12% using non-expert evaluators. This may represent a methodological advance by demonstrating that neurophysiological information derived from EEG can be perceptually discriminated through sonification alone, without requiring specialized expertise or multimodal integration.

This comparison reveals several methodological implications. First, the sonification method achieves diagnostic accuracy comparable to more computationally sophisticated techniques that require technical expertise. Second, while machine learning methods typically require large training datasets and exhaustive cross-validation, the proposed sonification approach can be implemented directly without a prior training phase. Third, unlike traditional quantitative methods that extract specific predefined features (spectral power, coherence, entropy), sonification preserves the complete temporal structure of the signal, allowing the human perceptual system to integrate multiscale information naturally.

However, it is important to recognize that the inter-rater variability observed in this study is considerably greater than the typical variability of automatic machine learning algorithms, which produce deterministic and reproducible classifications. This difference suggests that future iterations of the method could benefit from hybridizing both approaches: using sonification as an accessible initial screening tool, followed by automated quantitative analysis in ambiguous cases or for definitive diagnostic confirmation.

### 4.1. Methodological Implications

#### Considerations on Temporal Fidelity vs. Perceptual Discriminability

The parametric sonification method used transforms temporally aggregated spectral characteristics (band power, entropy) into perceptually distinguishable sound textures by mapping them to controllable synthesis parameters. Unlike direct amplitude-to-amplitude mapping, which preserves the exact temporal morphology by linearly converting EEG samples to sound pressure levels but produces sounds with limited timbral differentiability [20,21], the parametric approach sacrifices sample-to-sample correspondence in favor of maximizing perceptual discriminability by exploiting psychoacoustic principles of timbral segregation [15,27].

This strategy is particularly appropriate for the objective of the study, perceptual evaluation by evaluators without technical training, given that the neurophysiological differences between Alzheimer’s patients and healthy controls are predominantly manifested in the spectral domain [increased delta/theta power, decreased alpha/beta; [7,8]], characteristics that parametric mapping translates into global timbral differences that are perceptible through quantifiable variations in sound “color”: Alzheimer’s sonifications exhibit a downward shift in the spectral centroid toward lower frequencies, increased aperiodic and high-distortion components, and greater temporal modulation, whereas healthy controls display higher spectral energy in the mid-high range, lower harmonic distortion, and more temporally stable amplitude envelopes.

The trade-off between temporal fidelity and perceptual discriminability is a fundamental consideration in sonification design [47]. Our approach prioritizes human perception over exact signal preservation, justified by three reasons: (1) slow brain oscillations of clinical interest (delta: 1–4 Hz, theta: 4–8 Hz) are infrasonic, requiring transformation for effective perception; (2) evidence indicates that alterations in AD are predominantly spectral rather than transient morphological [10]; (3) the human auditory system better discriminates between different timbres than subtle variations in amplitude in narrowband signals [28].

However, alternative approaches to direct sonification with temporal compression could preserve more information about rapid transient dynamics (microstates, phase transitions), representing a complementary direction for future studies investigating temporal patterns of microstates or transient synchronization in Alzheimer’s EEG.

### 4.2. Inter-Evaluator Variability

The variability observed in classification accuracy between evaluators (range: 49.25–97.01%, SD = 17.95%) is a relevant finding that warrants detailed analysis. This heterogeneity suggests substantial individual differences in the ability to discriminate between sonified electroencephalographic patterns.

Two evaluators (4 and 5) did not achieve statistically superior performance to chance (*p* = 0.596 and *p* = 0.221, respectively), while six evaluators significantly exceeded this threshold, with three of them (6, 7, 8) achieving accuracies above 94%. This bimodality in performance suggests that auditory discrimination ability may not be universal, but rather depends on cognitive, perceptual, or strategic factors that we have characterized.

Several factors could explain this variability. First, differences in temporal auditory sensitivity could allow some evaluators to detect subtle fluctuations in the amplitude or frequency of the sonifications that others do not perceive. Second, heterogeneous perceptual strategies (e.g., focus on global vs. local characteristics, attention to temporal vs. spectral patterns) could generate systematic divergences. Third, differences in auditory working memory capacity could affect the integration of information across files lasting several minutes.

This heterogeneity has important implications for future applications. Identifying the cognitive and perceptual factors that predict success in auditory discrimination would enable the development of specific training protocols, perceptual assessment batteries to select optimal evaluators, or even computer support systems that amplify the most discriminative acoustic characteristics.

### 4.3. Advantages of the Proposed Approach

The method developed has a number of key advantages that reinforce its applicability in both clinical and experimental settings. First, it stands out for its accessibility, as it does not require advanced technical knowledge for an initial interpretation of the results, making it easy to use by professionals from different disciplines. In addition, it is remarkably cost-effective: the procedure is based on the use of standard EEG equipment and relatively simple computational processing, without the need for complex infrastructure or proprietary software.

Another relevant feature is its speed, as auditory evaluation enables an immediate appraisal of brain patterns, substantially reducing analysis time compared to traditional quantitative approaches. Likewise, the method stands out for its intuitiveness, leveraging the natural perceptual capacities of the human auditory system. Finally, its complementarity constitutes an added value, as it can be integrated synergistically with other diagnostic and analytical techniques.

This method represents a contribution to the development of accessible and cost-effective diagnostic tools. The non-invasive nature of EEG, combined with the simplicity of the proposed processing pipeline, could facilitate the development of affordable screening systems for the early detection of AD in resource-limited settings [48,49].

Precisely, one of the most significant aspects of this methodological approach is its potential to explore complementary methods that facilitate access to Alzheimer’s biomarkers. Traditionally, the most specific biomarkers of AD, such as positron emission tomography with amyloid or tau tracers, and cerebrospinal fluid biomarker analysis, remain concentrated in research institutes and specialized tertiary hospitals, making them inaccessible to most of the population, particularly in rural areas and in low- and middle-income countries [50,51]. EEG, by contrast, represents a low-cost, portable, and widely available technology that does not require complex hospital infrastructure.

The proposed methodology enhances this accessibility by radically simplifying the interpretation process. Whereas traditional quantitative EEG analysis requires specialized software, advanced neurophysiological expertise, and intensive computational processing, our direct sonification approach transforms complex signals into intuitive auditory representations that can be evaluated with minimal equipment and without specialized technical training. The demonstrated proof-of-concept suggests that parametric sonification preserves discriminative neurophysiological information in a perceptually accessible form. Future research could explore whether this approach, following extensive validation, might eventually contribute as a complementary tool within multimodal diagnostic frameworks. However, such potential applications would require prospective multicenter validation, assessment of diagnostic reliability in independent populations, cost-effectiveness analyses, and implementation studies addressing practical, regulatory, and ethical barriers to clinical adoption.

### 4.4. Limitations of the Study and Future Directions

This study presents several limitations that should be considered when interpreting the results. Although the dataset used (n = 65) is appropriate for a feasibility or proof-of-concept study, larger cohorts are required for robust clinical validation. Additionally, the data originate from a single institution and geographic population, which may limit the generalizability of the findings. Independent datasets are also needed to confirm the reproducibility of the method. The study does not address individual variability in auditory discrimination abilities among evaluators. It should also be considered that the sonification may contribute solely to differentiating healthy from pathological brain activity. These issues should be taken into account in future developments.

The parametric mapping approach based on 2 s windows involves temporal aggregation that may obscure fast transient dynamics (EEG microstates, phase transitions, transient synchronization). Complementary methods that preserve microstructural temporal information could capture additional relevant patterns.

On the other hand, the present work focused on local spectral features by region, without evaluating measures of inter-regional synchronization (coherence, phase-locking value, transfer entropy). Incorporating functional connectivity parameters in future developments could enrich the sonic representation of altered brain network patterns in AD.

The heterogeneity in individual evaluator performance (range: 49.25–97.01%) represents an important methodological limitation that warrants further investigation. Specifically, two of the eight evaluators did not achieve performance levels statistically above chance, which constrains the generalization of the findings regarding the method’s universal feasibility. This variability suggests that uncontrolled individual factors (auditory sensitivity, perceptual strategies, sustained attention capacity) may significantly moderate the effectiveness of auditory discrimination.

Additionally, the relatively limited size of the evaluator panel (n = 8) and the set of assessed subjects (n = 65) restricts the statistical power to detect subtle effects and limits the generalizability of the observed inter-rater agreement patterns. Future studies with larger samples that are geographically and culturally diverse will be necessary to confirm the replicability of these findings.

Finally, the exclusion of 4 subjects from the inter-evaluator agreement analysis (61 of 65 items used for Kappa), due to incomplete or ambiguous classifications, introduces potential selection bias. It is possible that these cases represent precisely the most difficult to classify, and their exclusion may artificially inflate the agreement indices reported.

An additional methodological consideration concern the sample size relative to the number of evaluators. While n = 65 is appropriate for exploratory proof-of-concept studies, it is relatively limited for robust estimation of individual diagnostic reliability, particularly for evaluators performing near chance levels. The observed heterogeneity (range: 49.25–97.01%) suggests that larger samples would better characterize the distribution of evaluator abilities. Furthermore, the current design does not allow assessment of intra-rater reliability (test–retest), as each evaluator heard each sonification only once. Future studies should employ larger samples (N > 100) and repeated evaluations to more robustly characterize individual performance and determine minimum sample sizes required for reliable diagnostic assessment.

It is important to emphasize that this study constitutes a methodological proof of concept demonstrating that discriminative EEG information can be preserved through parametric sonification and perceived auditorily. However, it does not provide evidence regarding the generalizability of these findings to real clinical contexts, the diagnostic reliability in independent populations, the practical applicability in primary care settings, or the effectiveness of this approach as a population-level screening tool. The clinical translation of this method requires prospective multicenter validation, assessment of its predictive value in independent cohorts, cost-effectiveness analyses, and implementation studies that address the practical, regulatory, and ethical barriers to its clinical adoption.

Another limitation of the present study is that evaluators performed binary classifications without providing confidence ratings or probabilistic estimates. While the reported metrics (accuracy, sensitivity, specificity, inter-rater agreement) appropriately characterize performance in binary forced-choice tasks, future studies could enrich the analysis by collecting confidence ratings alongside classifications (e.g., “How confident are you that this subject has AD?” on a 0–100% scale). This would enable signal detection theory analysis (calculation of d’ and criterion c) [35,36] or construction of ROC curves that would provide finer-grained characterization of perceptual sensitivity, response bias, and individual decision thresholds. Additionally, collecting verbal protocols during evaluation (“think-aloud”) could reveal the specific perceptual strategies employed by high-performing evaluators, facilitating the development of structured training programs to improve inter-rater reliability.

The results obtained open several promising lines of research. First, it will be essential to advance algorithmic optimization by developing more sophisticated sonification methods that maximize auditory discriminability between groups. In this regard, systematically exploring the individual factors that make a person a better perceiver of sonic differences would help inform the creation of targeted training programs for evaluators. In parallel, multimodal integration with other non-invasive biomarkers could increase diagnostic accuracy and enhance the robustness of the proposed approach.

Likewise, there is a need for longitudinal studies evaluating the method’s ability to detect changes associated with disease progression over time. Another relevant direction would be the automation of the process through the development of systems capable of automatically recognizing sonified patterns, which would enable a more objective and reproducible interpretation. An important aspect, given that this study focused solely on binary discrimination between AD and healthy controls, will be the inclusion of additional pathologies to clarify whether sonification is sensitive to specific disease processes or simply highlights perceptible differences between pathological and healthy dynamics. Finally, advancing toward clinical validation will be essential, implementing the method in real healthcare settings in order to assess its practical utility and potential impact on the early diagnosis of neurodegenerative diseases.

## 5. Conclusions

This study demonstrates that parametric EEG sonification preserves discriminative neurophysiological information that is perceptible through auditory inspection, enabling the differentiation between patients with AD and healthy controls. The accuracy of 76.12% achieved by evaluators with no prior EEG experience far exceeds chance level, indicating that the human auditory system possesses an inherent sensitivity to subtle alterations in spectral dynamics associated with neurodegeneration.

The combination of this perceptual capacity with the accessibility and low cost of EEG provides a promising framework for the development of complementary diagnostic tools. In this context, this proof-of-concept study demonstrates that parametric EEG sonification can preserve discriminative neurophysiological information accessible through auditory perception. While the method is technically simple and could theoretically be implemented with standard equipment, extensive further validation is required before any consideration of clinical application. Future research should address generalizability, diagnostic reliability in independent populations, and integration into multimodal diagnostic frameworks. By transforming complex EEG analyses into accessible auditory evaluations, direct sonification could offer a methodological foundation for future research on auditory-based diagnostic screening strategies.

Finally, the clinical implications include its potential use as a screening tool, as a complement to neuropsychological assessments, or as a system for monitoring disease progression. However, additional studies with larger samples and clinical validation are required to confirm its practical applicability. These results lay the groundwork for future research in the emerging field of neurological sonification, at the intersection of computational neuroscience, auditory perception, and digital medicine.

## Figures and Tables

**Table 1:** Demographic and clinical characteristics of participants.

Characteristic	Alzheimer’s Group (n = 36)	Control Group (n = 29)
Age, years, M (SD)	66.4 (7.9)	67.9 (5.4)
MMSE, M (SD)	17.75 (4.5)	30 (–)
Duration of illness, months, median (IQR)	25 (24–28.5)	—

Note. M = mean; SD = standard deviation; IQR = interquartile range; MMSE = Mini-Mental State Examination.

**Table 3:** Performance Metrics by Evaluator in the Auditory Classification Task.

Evaluator	Accuracy (%)	Sensitivity (%)	Specificity (%)	PPV (%)	NPV (%)	*p*-Value *	95% CI
1	74.63	79.41	82.14	84.38	76.67	<0.001	[63.1, 83.5]
2	68.66	66.67	75.86	77.42	64.71	0.005	[56.8, 78.5]
3	71.64	69.44	82.14	83.33	67.65	0.001	[59.9, 81.0]
4	49.25	47.22	55.17	56.67	45.71	0.596	[37.6, 60.9]
5	58.21	47.22	75.86	70.83	53.66	0.222	[46.3, 69.3]
6	94.03	94.44	100.00	100.00	93.55	<0.001	[85.6, 97.7]
7	95.52	94.59	100.00	100.00	93.55	<0.001	[87.6, 98.5]
8	97.01	97.30	100.00	100.00	96.67	<0.001	[89.8, 99.2]
Mean	76.12	74.54	83.90	84.08	74.02	—	—
SD	17.95	20.43	15.76	15.73	19.37	—	—

Note. PPV = Positive Predictive Value; NPV = Negative Predictive Value; 95% CI = 95% Confidence Interval; SD = Standard Deviation. * *p*-values adjusted using Holm correction for multiple comparisons. Global permutation test: *p* = 0.001. Statistical power: 0.916. N = 65 subjects (36 Alzheimer’s, 29 controls).

**Table 4:** Summary of Overall Metrics and Inter-Evaluator Agreement.

Metric	Value	95% CI
Average accuracy (%)	76.12	—
Standard deviation (%)	17.95	—
Accuracy range (%)	49.25–97.01	—
Fleiss’ Kappa	0.511	[0.370, 0.636]
Permutation test (p)	0.001	—
Statistical power	0.916	—
N evaluators	8	—
Total N subjects	65	—
Items N used (Kappa analysis)	61	—

Note: 95% CI = 95% confidence interval. The permutation test evaluated the probability of obtaining an average accuracy equal to or greater than that observed under the null hypothesis of random classification. The Kappa coefficient indicates moderate agreement according to the Landis and Koch scale (1977) [40]. The accuracy range reflects the minimum (4) and maximum (8) individual performance of the evaluators.

**Table 5:** Objective Acoustic Differences Between AD and Control Sonifications.

Acoustic Descriptor	AD Mean ± SD	Control Mean ± SD	Difference	t-Statistic	*p*	Cohen’s d
Energy 90–170 Hz (%)	27.74 ± 9.51	15.79 ± 4.62	+11.95	6.11	<0.001 ***	1.55
Energy 180–340 Hz (%)	12.29 ± 7.53	21.22 ± 6.92	−8.92	−4.85	<0.001 ***	−1.23
Energy 600–2000 Hz (%)	3.99 ± 1.36	5.68 ± 0.89	−1.69	−5.66	<0.001 ***	−1.43
Spectral centroid (Hz)	4062 ± 995	4822 ± 868	−760	−3.19	0.004 **	−0.81
Energy < 100 Hz (%)	33.35 ± 8.49	27.50 ± 6.07	+5.85	3.07	0.004 **	0.78
Energy > 2000 Hz (%)	31.43 ± 7.13	36.74 ± 6.78	−5.31	−3.00	0.005 **	−0.76
Temporal CV	0.312 ± 0.045	0.354 ± 0.035	−0.042	−4.07	<0.001 ***	−1.03
Harmonicity estimate	1.15 ± 0.49	0.77 ± 0.20	+0.38	3.83	<0.001 ***	0.97

Note: All *p*-values corrected using FDR (Benjamini–Hochberg). ** *p* < 0.01, *** *p* < 0.001.

**Table 6:** Comparison of the Diagnostic Accuracy of Different Electroencephalographic Biomarkers in AD.

EEG Biomarker	Diagnostic Accuracy	Main Characteristics	Reference
Quantitative spectral analysis (band power)	70–85%	Analysis of relative power in delta, theta, alpha, and beta bands. Requires specialized technical expertise.	Dauwels et al., [8]
Functional connectivity analysis	75–90%	Evaluation of coherence and synchronization between brain regions. Computationally intensive.	Stam et al., [11]; Rossini et al., [10]
Machine learning with EEG features	78–92%	Automatic classifiers (SVM, Random Forest) trained on multiple spectral and temporal features.	Trambaiolli et al., [44]
Signal complexity analysis (entropy)	72–88%	Measures of spectral entropy, sample entropy, and Lempel–Ziv complexity.	Horvath et al., [26]
Parametric sonification with perceptual evaluation (current study)	76.12%	Frequency-domain spectral analysis. Real-time parametric synthesis. Auditory evaluation requiring no technical expertise. Accessible and low-cost method.	Present study

Note. SVM = Support Vector Machine. The accuracy ranges reported correspond to meta-analyses and systematic reviews of multiple studies. The accuracy of the present study represents the average of 8 evaluators with no prior experience in EEG interpretation (95% CI = [60.7%, 86.3%]).

## Data Availability

The data that support the findings of this study are publicly available on Zenodo: Dataset DOI: 10.5281/zenodo.17571308.

## Note

This work is part of Rubén Pérez-Elvira’s Ph.D. Thesis in medicine and surgery.
